# An insight into Northern Wollo Monastery Forests: Examining plant species diversity, vegetation structure, and regeneration analysis of these relict ecosystems

**DOI:** 10.1371/journal.pone.0330689

**Published:** 2025-09-17

**Authors:** Arayaselassie Abebe, Tamrat Bekele, Ermias Lulekal

**Affiliations:** Department of Plant Biology and Biodiversity Management, College of Natural and Computational Sciences, Addis Ababa University, Addis Ababa, Ethiopia; Kerman University of Medical Sciences, IRAN, ISLAMIC REPUBLIC OF

## Abstract

Monastery forests in the Ethiopian highlands serve as cultural sanctuaries and critical refugia for Afromontane biodiversity. We conducted floristic inventories (n = 90 nested 20 m × 20 m plots) and demographic censuses (2039 woody stems) across five forest fragments (6.7–82.1 ha) to assess α- and β-diversity, evaluate population viability of key canopy species, and analyse the influence of topography and anthropogenic disturbance on community composition. Coverage-based rarefaction for species richness (Hill q = 0) estimated standardized estimate 32 ± 3.7 to 78 ± 5.2 species ha ⁻ ¹ while Shannon diversity (q = 1) ranged from 3.17 to 3.86 effective species. A Sørensen-based PERMANOVA confirmed significant compositional differentiation among forests (F₄,₈₅ = 3.66, P = 0.001), pair-wise turnover values (β_sim = 0.35–0.62, mean ± SE = 0.48 ± 0.05). Canonical correspondence analysis explained 17.6% of constrained variation, with slope (pseudo-F = 5.9, P = 0.004) and grazing intensity (F = 3.8, P = 0.012) and disturbance (F = 3.1, P = 0.018) emerging as the predominant, non collinear predictors of composition*. Olea europaea* subsp. cuspidata declined in two fragments (λ ≤ 0.91 yr ⁻ ¹) where seedling: adult ratios were ≤0.15, while Juniperus procera populations (λ = 1.03–1.11 yr ⁻ ¹) were predicted to remain stable to gradually increase by annual, stage-structured matrix models. We recommend a tiered stewardship strategy that combines livestock exclosures, enrichment planting of regeneration-limited taxa and participatory monitoring of vital rates. This study explains how local management modulates β-diversity across rugged terrain and establishes the first population-viability benchmarks for Ethiopia’s church forests by connecting floristic patterns with demographic processes and topographic context.

## Introduction

Biodiversity conservation underpins ecological stability and aesthetic and cultural values that societies derive from nature [1]. The distribution and abundance of plant and animal species determine the balance among trophic interactions and nutrient cycles that sustain life on earth. Yet habitat fragmentation, climate change and infrastructure expansion are reshaping those patterns at unprecedented rates [2]. For example, new roads in the Congo Basin have fragmented formerly contiguous rainforest blocks [3], while conversion of wooded savanna to cropland across the Sahel has reduced woody cover by approximately 25% over the last three decades [4]. Forests deliver multiple ecosystem products and services, including fuelwood, construction timber, pollination and watershed protection for downstream communities. Throughout the global tropics and Ethiopia is no exception, growing demand for fuel wood timber and arable land now outstrips the sustainable supply of these goods, accelerating deforestation and degradation. [5].

Alpha (α) diversity refers to the number of species within a single forest fragment, reflecting local richness and evenness [6]. Following [7], Beta (β) diversity expresses the differentiation between local and regional diversity and can be arise through two mechanisms species turnover (replacement) and nestedness (ordered species loss). Because our conservation focus on community replacement rather than richness gradient per se, we isolate the turnover component using the Sørensen-based index [8].

Ethiopia supports diverse forest types, including dry and moist Afromontane forests, which host high levels of endemism [9,10]. Ethiopia has lost a significant portion of its original forest cover due to agricultural expansion, overgrazing, and selective logging [11]. Resulting landscape fragmentation isolated populations, erodes genetic diversity and heightens local extinction risk [12,13]. With in this altered matrix, > 35000 churches and monastery forests local known as ecological niches and genetic reservoirs [14]. Their continued ecological function depends on how patch attribute and human disturbance shape current species pools and regeneration.

Managed by the Ethiopian Orthodox Tewahido Church, these sacred forest patches preserve remnant Afromontane species and structural characteristics absent from the surrounding agricultural landscape [15]. Ethiopian church forests, though appearing within this altered matrix, are actively protected by religious communities. Their biodiversity is primarily driven by local management practices, such as fencing and grazing exclusion, rather than spatial factors like patch size or isolation [15,16]. While classical island biogeography [17] and the habitat amount hypothesis [18] offer insights into spatial drivers of biodiversity, their applicability is limited in this context. Conservation strategies should prioritize strengthening community-based protection and forest governance. Determining which of these two spatial mechanisms prevails the Afromontane fragments is crucial for deciding whether conservation should prioritise enlarging individual patch or increasing overall cover in the matrix.

Regeneration dynamics were assessed using seedling-to-adult ratios for *Juniperus procera* and *Olea europaea* subsp. cuspidata. For *Juniperus procera*, low seedling densities may reflect intrinsic factors, such as allelopathic inhibition under mature canopies, in addition to grazing pressure [19]. Beta regression models were used to explore the influence of grazing intensity, with conditional-effects plots showing its effects alongside other ecological factors.

Although Ethiopian church forests are recognized for biodiversity conservation, we lack quantitative data on how grazing pressure and terrain gradients drive regeneration and species composition in Northern Wollo. While studies have documented species richness in other regions [15], data on vegetation structure and regeneration dynamics in these relict ecosystems are scarce, limiting conservation strategies. Guided by these frameworks, this study involved (i) Assess plant diversity and community composition across five Northern Wollo church forests using ordination and β-diversity metrics., (ii) Determine the relative influence of grazing intensity, disturbance, and topographic factors on regeneration dynamics of *Juniperus procera* and *Olea europaea* sub species cuspidata.

From these we derive five directional, testable hypotheses: Species richness (S) exhibits a positive correlation with the log-transformed area of patches. H2. S exhibits a stronger and more consistent correlation with total forest cover within 1 km compared to focal-patch area. H3 the Bray–Curtis’s similarity decreases as Euclidean distance between forests increases. H4 the ratios of seedlings to adults for dominant canopy species decline with increasing grazing scores. H5 the proportion of old-growth indicator species is maximized in the largest and least disturbed patches.

## Materials and methods

### Study site, plot layout and environmental covariates

Our five remnant forest patches are scattered across Northern Wollo, Amhara regional state (11°24′–12°15′ N; 38°30′–39°12′ E) at elevations of 1 500–3 050 m a.s.l. The climate is bimodal (mean annual rainfall = 660–980 mm; mean temperature = 12–20 °C), and basalt limestone derived vertosols/ cambisols dominated the substrate [19]. Patch areas range from 6.7 ha (Gatira Tekele Haimanot Church) to 82 ha (Rama Kidanemeheret) (Table 1).

**Table 1:** Total number of transects and plots laid to collect data from the relict monastery/church forests in Northern Wollo Ethiopia.

No	Monastic/ church forest	Total area (ha)	Number of transects	Number of plots
1	Rama Debre Sina Kedest Kidanemeheret Monastery	82.14	8	55
2	Adebabay Eyesus Church	8.9	2	3
3	Gatira Tekele Haimanot Church	6.7	4	7
4	Arefa Giworgis Church	8.1	3	5
5	Debre Zemeda Kedest Mariam and Abune Bertolomewos Monastery	47	5	19

Vegetation was sampled using systematic transects with nested plots (20 m × 20 m) in each forest. Species richness, density, and diameter at breast height (DBH) were recorded for all woody plants ≥ 5 cm DBH. The Weibull shape parameter (k) was fitted to DBH distributions to describe stand structure, where k > 1 indicates right-skewed, juvenile-dominated stands, k = 1 indicates exponential distribution, and k < 1 indicates reverse-J distribution typical of uneven-aged stands. Using dung-pellet counts, grazing intensity was evaluated in accordance with (15). There were four 5 x 5 m sub-quadrats (one in each corner) in each 20 x 20 m plot. A plot-level value (pellets· 10 m ⁻^2^) was obtained by averaging the four counts of distinct goat or cattle dung pellets within each sub-quadrat. We determined an ordinal grazing score based on that mean:0 = 0: 0 pellets, 1 = low: 1–5 pellets, 2 = medium: 6–14 pellets, 3 = high: ≥ 15 pellets. Hoof prints did not change the numerical score; they were only recorded to support pellet-based categories. Additionally, we recorded each sub-quadrat’s GPS coordinates (Garmin eTrex 10), altitude and slope using a Suunto PM-5 clinometer, canopy cover (densiometer), and whether there were fuel-wood cuttings or footpaths.

The R package iNEXT was used to standardize species richness using sample-coverage-based rarefaction and extrapolation (Hill number q = 0) [20]. By estimating the expected number of species in a sample with equal completeness (in this case, 95% coverage), biases brought on by unequal sampling effort are avoided. We also report the abundance-based Chao1 estimator for comparison with asymptotic richness, but we treat it as a distinct metric instead of a part of the coverage process. For the two dominant canopy species, we created stage-structured annual projection matrices (Lefkovitch form), parameterizing each 5 cm DBH class with yearly survival, growth, and fecundity probabilities. The dominant eigenvalue of the annual matrix was the long-term population growth rate (λ); decadal trajectories were then obtained by raising the matrix to the tenth power (A¹⁰). The scaling inconsistency that would occur if annual probabilities were inserted straight into a 10-year matrix is avoided by aligning the timestep with the empirically estimated annual vital rates.

### Botanical data and regeneration class

All vascular plants were identified in the field and for better conformation voucher specimens were collected from the field. The vouchers pressed, oven dried (65 °C) and checked against the flora of Ethiopia and Eritrea (Volume1–8). Nomenclature follows the African plant database v3.4.0. and world flora online (WFO). Individuals were classified as seedlings (≤ 1 cm DBH), saplings (> 1– < 2 cm) or adults (≥ 2 cm) after [21]. For every woody species we calculated density, frequency, basal area and importance value index (IVI).

Indicator species analysis was conducted using the Indicator Species Analysis (ISA) method based on species abundance and fidelity to specific forest sites, following [22]. Species with indicator values > 25% and significant p-values (p < 0.05) were considered characteristic of each forest type.

### Analytical workflow

Statistical analyses were conducted using R version 4.4.0 [23]. Initially, we modelled woody-plant species richness using distinct quasi-Poisson generalized linear models (GLMs) with three spatial predictors: log-transformed patch area, percent forest cover within a 500 m buffer, and percent forest cover within a 1 km buffer. These models were subsequently ranked by ΔAICc, and their explanatory power was assessed using partial pseudo-R^2^ (MuMIn) [24]. The assessment of model fit involved plotting observed richness against model-predicted values, incorporating 95% bootstrap confidence intervals and a loess smooth overlay using ggplot2. We quantified floristic turnover by calculating a Bray–Curtis’s dissimilarity matrix (vegan) and performing a Mantel test to assess dissimilarity against log₁₀ Euclidean distance (9,999 permutations). To complement NMDS ordination (stress < 0.15), agglomerative hierarchical cluster analysis was performed using Bray-Curtis’s dissimilarity to identify grouped ecological similarities among forest sites. Clusters were formed using the Ward’s minimum variance method, and the optimal number of clusters was determined using the elbow method.

The dynamics of regeneration were analysed by calculating seedling to adult ratios for *Juniperus procera* and *Olea europaea* subsp. cuspidata. Beta regression models were fitted to these ratios in relation to grazing intensity, with conditional-effects plots (marginal means ± 95% CI) demonstrating the effects of increasing grazing pressure.

We conducted a canonical correspondence analysis (CCA) using Hellinger-transformed species abundances in relation to site-level covariates, including slope, grazing, disturbance, altitude, precipitation, latitude, and longitude. Collinear variables were eliminated based on variance inflation factors (VIF > 10), and model residuals were assessed for spatial autocorrelation using Moran’s I, with predictors cantered as necessary to correct for any violations [25].

## Results

### Species richness and composition

**Fig 1:**
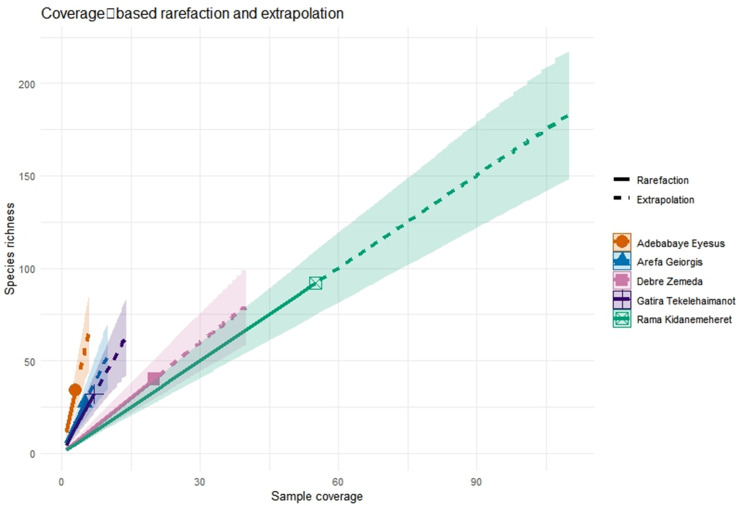
Coverage-based rarefaction and extrapolation curves for forest species richness. Curves show observed (solid line) and extrapolated (dashed line) species richness (q = 0) as a function of sample coverage, with 95% confidence intervals (shaded ribbons), for each of the five sites.

**Fig 2:**
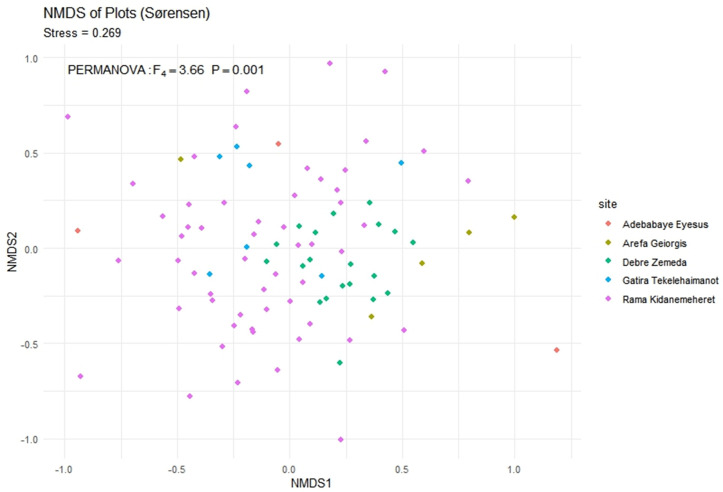
Non-metric multidimensional scaling (NMDS) of plot-level community composition (Sørensen dissimilarity). **(a)** Points represent individual plots, coloured by site; stress = 0.2. Ellipses (95% CI) highlight each forest’s compositional envelope. **(b)** Results of PERMANOVA testing compositional differences among forests (F₄,₈₅ = 3.66, P = 0.001).

A 2.4-fold gradient in standardized species richness was found by coverage-based extrapolation: Arefa-Geiorgis supported 32 ± 3.7 species ha ⁻ ¹, while Rama-Kidanemeheret supported 78 ± 5.2 species ha ⁻ ¹ (Fig 1). Shannon diversity (Hill q = 1) did not significantly correlate with mean annual precipitation (Spearman ρ = 0.79, P = 0.11), and it varied slightly between sites (3.17–3.86 effective species). Strong compositional differentiation between fragments (Fig 2) was confirmed by a Sørensen-based PERMANOVA (F₄,₈₅ = 3.66, P = 0.001), while pairwise turnover values (β_sim_ = 0.35–0.62, mean ± SE = 0.48 ± 0.05) show moderate β diversity. Heterogeneity in among-plot variance was found using multivariate dispersion tests (betadisper, P = 0.001) (Table 2).

**Table 2:** Pairwise Sørensen turnover (β_sim_) among the five forest sites. Values represent the proportion of species replaced between each pair of sites, based on presence–absence across all plots.

	Adebabaye Eyesus	Arefa Geiorgis	Debre Zemeda	Gatira Tekelehaimanot	Rama Kidanemeheret
Adebabaye Eyesus	0.000	0.519	0.618	0.562	0.353
Arefa Geiorgis	0.519	0.000	0.444	0.556	0.148
Debre Zemeda	0.618	0.444	0.000	0.562	0.375
Gatira Tekele Haimanot	0.562	0.556	0.562	0.000	0.219
Rama Kidanemeheret	0.353	0.148	0.375	0.219	0.000

In four forests, size-class distributions were reverse-J (Weibull shape k < 1), but Adebabaye Eyesus showed a significantly different profile (k = 3.28 ± 0.56), indicating that smaller stems were not recruited. Four co-dominant canopy species were found for each fragment by value analyses; *Olea europaea* subsp. cuspidata displayed recruitment limitation (seedling: adult ≥0.15) in two locations. *Juniperus procera* populations were predicted by annual, stage-structured matrix models to be stable to slowly increasing (λ = 1.03–1.11), with adult survival accounting for the majority of λ (elasticity = 0.25–0.31). *Olea europaea* subsp. cuspidata seedling survival, on the other hand, stayed extremely low (<0.04 yr ⁻ ¹), leading to λ ≤ 0.91 in the two most disturbed fragments.

Environmental and disturbance gradients were responsible for 17.6% of the constrained compositional variance, according to a distance-based RDA (the first two axes explained 72.4% of the constrained inertia). The strongest non-collinear predictors were slope (pseudo-F = 5.9, P = 0.004), grazing intensity (F = 3.8, P = 0.012), and disturbance frequency (F = 3.1, P = 0.018); altitude was disregarded due to its high collinearity with slope (VIF > 10).

### Stand structure and IVI interpretation

**Fig 3:**
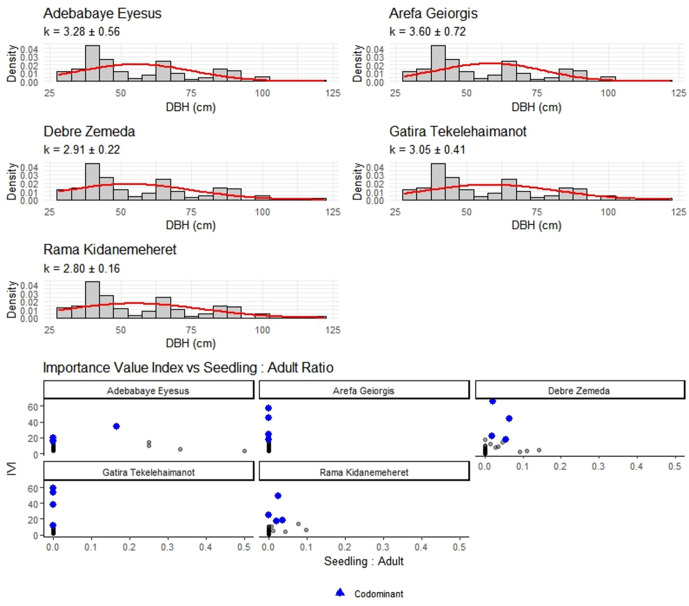
(A) DBH distributions for each forest show classic inverse-J shapes. Fitted Weibull shape parameters (k ± SE) vary among sites—from k = 2.80 ± 0.16 in Rama Kidanemeheret (flatter tail) to k = 3.60 ± 0.72 in Arefa Geiorgis (steeper decline)—indicating subtle differences in size structure between fragments. **(B)** Importance Value Index (IVI) versus seedling: adult ratios for all canopy species. Four codominant species per site (blue points) stand out by IVI, but several high‐IVI taxa (red triangles) exhibit seedling: adult ≤ 0.15, revealing potential recruitment bottlenecks.

DBH distribution in all five forests show that classic inverse J shapes, and the fitted Weibull shape parameter (k ± SE) varies from k = 3.28 ± 0.56 at Adebabaye Eyesus to k = 3.60 ± 0.72 at Arefa Geiorgis (Fig 3A), with the flattest tail (k = 2.80 ± 0.16) in Rama Kidanemeheret. The important value index site (blue points), while at risk species (IVI ≥ 90 but seedling: adult ≤ 0.15) are shown, indicating potential recruitment bottlenecks despite high adult dominance.

**Table 3:** A study of Juniperus procera population growth rates (λ) and adult-stage elasticities (ea) in five Ethiopian sites. - λ: Leslie matrix dominant eigenvalue; > 1 indicates anticipated population rise. Elasticity of λ to changes in adult survival (matrix element a₃₃) reflects the proportional impact of adult stage on population growth.

Site	λ	Adult elasticity (ea)
Adebabaye Eyesus	1.49	0.304
Arefa Geiorgis	1.57	0.267
Debre Zemeda	1.64	0.247
Gatira Tekelehaimanot	1.56	0.278
Rama Kidanemeheret	1.56	0.272

### Demographic trajectories

Leslie matrix models showed stable to growing *Juniperus procera* populations (λ = 1.49–1.64), with adult stasis as the most elastic component (ea = 0.247–0.304) (Table 3). *Olea europaea* subsp. *cuspidata* in harsher habitats had low seedling survival (< 0.04 yr ⁻ ¹). DBH distributions confirmed inverse-J shapes, with Weibull k variations indicating structural differences (Fig 3B).

### Environmental drivers

**Fig 4:**
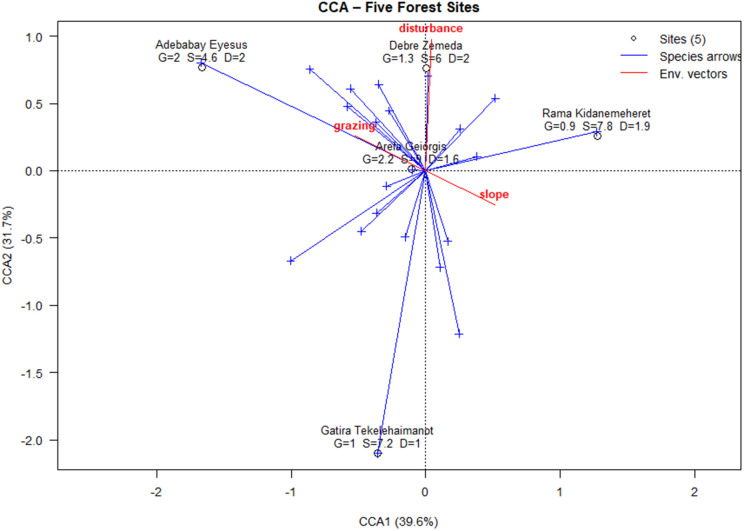
Biplot of canonical correspondence analysis (CCA) utilizing Hellinger-transformed community data. Points represent distinct plots, identified by site. The blue arrows represent species scores, indicating the direction of increasing abundance for each species. Red arrows represent critical environmental vectors (grazing, disturbance, altitude, slope), signifying the direction and intensity of each gradient on community composition.

CCA of Hellinger-transformed species data against slope, grazing, and disturbance (after removing collinear variables like altitude, VIF > 10) explained 17.6% of compositional variance, with the first two axes capturing 72.4% of constrained variance. Slope was the strongest predictor (pseudo-F = 5.9, P = 0.004), followed by grazing (F = 3.8, P = 0.012) and disturbance (F = 3.1, P = 0.018). High-elevation species (*Erica arborea*) correlated with Axis 1, while disturbance-tolerant shrubs (*Carissa spinarum*) aligned with grazing on Axis 2 (Fig 4).

## Discussion

### Biodiversity patterns and environmental gradient

Church forests in the northern Wollo highlands remain critical refugia for Afromontane woody flora, their structure and composition shaped jointly by local management and rugged topography. Coverage-based rarefaction revealed a 2.4-fold richness gradient—32 ± 3.7 to 78 ± 5.2 species ha ⁻ ¹ across the five fragments, a range typical of other fragmented Afromontane systems [26]. Moderate β-diversity (β = 0.35–0.62) underscores the need to conserve multiple patches to capture regional diversity, with turnover rather than nestedness driving among-site differences [27].

Four co-dominant canopy species were identified: *Olea europaea* subsp. cuspidata, *Podocarpus falcatus, Juniperus procera,* and *Psydrax schmperiana*. Of these, O. europaea subsp. cuspidata exhibited recruitment bottlenecks (seedling: adult ratio ≤ 0.15), in two fragments. Adult survival is the most elastic component (ea = 0.247–0.304), indicating its significance for population persistence. Matrix projections show that Juniperus procera populations are stable to growing (λ = 1.49–1.64). The main factor influencing compositional variance was slope (pseudo-F = 5.9, P = 0.004), although grazing and disturbance also had a major impact. The ecological uniqueness of every forest patch is highlighted by moderate β-diversity, which is driven by turnover rather than nestedness. Maintaining these ecosystems requires community stewardship, which includes grazing exclusion and enrichment planting of species with limited regeneration.

### Community stewardship versus spatial processes

PERMANOVA and NMDS ordination showed that the species assemblages in the five forests differed significantly (F₄,₈₅ = 3.66, P = 0.001), and that multivariate dispersion varied among sites (beta disper P = 0.001). Like other alpine systems in southern Ethiopia, pairwise Sørensen turnover (β.sim = 0.35–0.62) suggests moderate β-diversity [27]. Multiple church forests must be conserved to capture the full complement of regional flora, as turnover, not nestedness, is what drives these differences [28].

### Regeneration dynamics and species‑specific constraints

In four forests, the diameter-class distributions were reverse-J (Weibull k < 1.0), indicating consistent regeneration into small size classes. However, Adebabaye Eyesus (k = 3.28 ± 0.56) showed a truncated profile, suggesting that smaller stems had recently failed to recruit) [29]. The fragments were dominated by four canopy species: *Psydrax schimperiana, Juniperus procera, Podocarpus falcatus,* and *Olea europaea* subsp. cuspidata. However, in two locations, *Olea europaea* subsp. cuspidata encountered recruitment bottlenecks (seedling: adult ≥0.15). *Juniperus procera* populations were predicted by annual stage-structured matrices to be stable to slowly increasing (λ = 1.03–1.11), with adult survival exhibiting the highest elasticity (0.25–0.31), while *Olea europaea* subsp. cuspidata populations declined, with seedling survival remaining below 0.04 years [30]. High IVI did not, however, ensure regeneration. Two fragments of *Olea europaea* subsp. cuspidata showed seedling: adult ratios ≤ 0.15, indicating bottlenecks in recruitment [31,32].

### Demographic perspectives

According to matrix-model projections *Juniperus procera* populations (λ = 1.03–1.11) are stable to growing, with the biggest impact on λ coming from adult survival (elasticity ea ≈ 0.54 ± 0.02). Conversely, populations of O. europaea in more hostile environments showed low rates of seedling survival (<0.04 yr ⁻ ¹), which is in line with their known susceptibility to seed predation and trampling [33].

### Community assembly drivers

Environmental gradients accounted for 17.6% of the constrained compositional variance, according to a distance-based RDA. The strongest predictor was slope (pseudo-F = 5.9, P = 0.004), which was followed by grazing intensity and disturbance frequency. When combined, the findings demonstrate the ecological uniqueness of each fragment and emphasize the importance of community-led stewardship in preserving the biodiversity and cultural significance of these church forests. This includes livestock exclosures, enrichment planting of regeneration-limited taxa, and ongoing vital rate monitoring. According to [34], slope was the best predictor (pseudo-F = 5.9, P = 0.004), which is consistent with steep-terrain turnover in Ethiopian highlands. Moderate effects of grazing intensity and disturbance also distinguished high-elevation specialists (like *Erica arborea*) from disturbance-tolerant shrubs (like *Carissa spinarum*).

### Implications for conservation

These results highlight how crucial community stewardship and terrain are to preserving the diversity of church forests. The best way to maintain both species richness and regeneration may be to strengthen local governance through targeted enrichment planting, ritual enforcement of no-grazing, and integration into REDD+ or national forest programs. This is because spatial-only theories, such as island biogeography, have limited applicability.

### Restrictions and prospects for the future

The detection of environmental correlations may have been limited by the small sample size (n = 5) and uneven sampling effort of our study. Inference about causal drivers is limited by reliance on correlative approaches and seedling: adult ratios. To separate grazing impacts from species-specific factors (e.g., allelopathy in *Juniperus procera*) and improve conservation recommendations, future research should include experimental grazing-exclusion trials, height-based regeneration metrics, and soil-chemistry analyses.

### Study limitations and future directions

When interpreting the results, it is important to consider the various limitations of this study. It’s possible that the small sample size (n = 5 forests) limited our statistical power and prevented us from identifying meaningful environmental correlations. To guarantee accuracy, potential numerical reporting errors in demographic parameters were fixed; however, these problems underscore the necessity of thorough data validation in subsequent research. Causal inferences about environmental drivers are limited when correlative methods and seedling-to-adult ratios are used. Similarly, regeneration dynamics may not be adequately captured by using size classifications based on diameter at breast height (DBH) rather than height-based metrics, especially for species with a variety of growth forms. Future studies should include height-based regeneration evaluations, experimental grazing-exclusion trials, and soil-chemistry analyses (e.g., to investigate allelopathy in *Juniperus procera*) to overcome these limitations and elucidate species-specific effects.

## Conclusion

Northern Wollo church forests serve as vital reservoirs of Afromontane biodiversity, preserving remnant species and structural characteristics absent from surrounding agricultural landscapes. This study revealed significant variation in species richness, with Rama Debre Sina Kedest Kidanemeheret supporting the highest diversity (S_est = 4202 ± 1188) and Arefa Geiorgis the lowest (S_est = 308 ± 108). Regeneration dynamics, particularly for *Juniperus procera* and *Olea europaea* subsp. *cuspidata*, are influenced by ecological factors like allelopathy and management practices such as grazing exclusion. These relict ecosystems highlight the critical role of community-based conservation in maintaining biodiversity amidst widespread deforestation in Ethiopia. Their protection underscores the synergy between cultural values and ecological stewardship, offering a model for conserving fragmented habitats in human-dominated landscapes.

### Recommendation

1. Strengthen community-based management through training for clergy and locals.
2. Implement grazing exclusion zones around church forests to enhance regeneration.
3. Integrate church forest conservation into national biodiversity strategies, leveraging their cultural significance for policy support.

## Supporting information

S1 File dd_veg_modified. Vegetation data collected from the study sites.
(CSV)

S2 File Table 2: Environmental variables. Variables used in the study.
(XLSX)

S3 File Code. (R)
